# Tools and analytic techniques to synthesise community knowledge in CBPR using computer-mediated participatory system modelling

**DOI:** 10.1038/s41746-020-0230-x

**Published:** 2020-02-19

**Authors:** Joshua Hayward, Saraya Morton, Michael Johnstone, Doug Creighton, Steven Allender

**Affiliations:** 1 Global Obesity Centre, Institute for Health Transformation, Deakin University, Geelong, 3220 Australia; 2 Institute for Intelligent Systems Research and Innovation, Deakin University, Geelong, Australia

**Keywords:** Obesity, Society

## Abstract

Participatory systems thinking methods are often used in community-based participatory research to engage and respond to complexity. Participation in systems thinking activities creates opportunities for participants to gain useful insights about complexity. It is desirable to design activities that extend the benefits of this participation into communities, as these insights are predictive of success in community-based prevention. This study tests an online, computer-mediated participatory system modelling platform (STICKE) and associated methods for collating and analysing its outputs. STICKE was trialled among a group of community members to test a computer-mediated system modelling exercise. The causal diagrams resulting from the exercise were then merged, and network analysis and DEMATEL methods applied to inform the generation of a smaller summary model to communicate insights from the participant group as a whole. Participants successfully completed the online modelling activity, and created causal diagrams consistent with expectations. The DEMATEL analysis was identified as the participant-preferred method for converging individuals causal diagrams into a coherent and useful summary. STICKE is an accessible tool that enabled participants to create causal diagrams online. Methods trialled in this study provide a protocol for combining and summarising individual causal diagrams that was perceived to be useful by the participant group. STICKE supports communities to consider and respond to complex problems at a local level, which is cornerstone of sustainable effective prevention. Understanding how communities perceive their own health challenges will be important to better support and inform locally owned prevention efforts.

## Introduction

Non-communicable diseases (NCDs) such as obesity, heart disease, cancer and diabetes have a number of modifiable risks that are influenced by a complex array of determinants working at multiple levels from individual choice through to environmental conditions and policy settings. Understanding this complexity has been a challenge to prevention in the past and has limited the effectiveness and sustainability of interventions.^1,2^ Post-normal methods that value pragmatic, constructivist approaches to addressing complexity are required to overcome the previous and limited rational, reductionist modes of research.^3^

Community-based participatory research (CBPR) is a post-normal approach to investigating complex issues, which values community participation and lived experience, builds capacity, and shares power between researchers and communities to support and empower action.^4^ The paradigmatic shift away from reductionism, toward a socioecological approach, and simultaneous focus on capacity building, co-learning and action strongly align the values of CBPR with those of systems thinking,^5^ which is operationalised through a wide variety of methods including system dynamics, network analysis (NA) and agent-based modelling among many others.^5,6^ Obesity prevention projects with CBPR underpinnings have incorporated participatory systems thinking activities into prevention strategy co-design efforts,^7–9^ with successes reported in both the generation of community action^10^ and longer-term health and behavioural outcomes.^11^

Community-level understanding of complex problems, and community attitudes toward potential solutions are predictive of intervention success in the community-based obesity prevention space,^12,13^ making them desirable outcomes for the broader community in a climate where systems thinking is increasingly being incorporated into population-level disease prevention. Tools that are accessible, and can facilitate system thinking activities are highly desirable as large, regional trials continue to develop and formalise the incorporation of systems thinking into CBPR.^14^

Informal system modelling exercises such as causal mapping confer useful insights about the multiplicity and interconnectedness of causes for complex problems.^15^ These exercises are amenable to delivery via computer-mediated platforms, so may facilitate accessible systems thinking activities that deliver useful insights, with the broad reach conferred by online platforms.

This paper reports on the initial testing of an online, computer-mediated participatory system modelling platform – Systems Thinking in Community Knowledge Exchange (STICKE), designed to support accessible participatory systems thinking activities relevant to the CBPR space.

The aims of this project were to:

1. Test STICKE as a computer-mediated tool to support a participatory system modelling activity for community-based users working remotely and individually, and:
2. Test different methods for analysing and presenting causal diagrams generated using the computer-mediated tool, to aggregate and present information to practitioners, researchers and policy makers.

## Results

### Completion of participatory system mapping activity

**Fig. 1:**
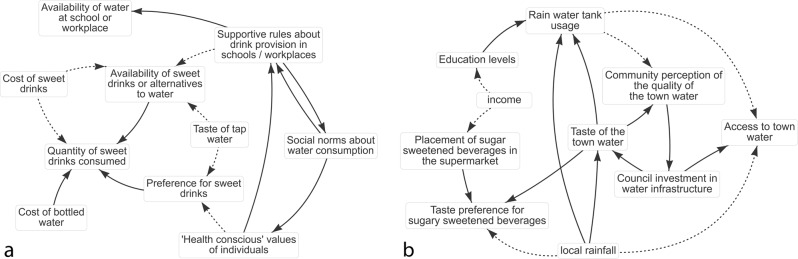
Examples of two individual diagrams from STICKE. Diagrams are composed of factors identified by the user as important causes of the problem under consideration (text boxes) and causal connections between them (arrows). Positive causal relationships are denoted by a solid arrow pointing from the cause to the effect. Inverse causal relationships are denoted by a dashed arrow pointing from the cause to the effect.

A total of 13 participants completed the test of the STICKE computer-mediated participatory system mapping activity. All participants were able to generate causal diagrams that were interpretable by the research team. The resulting diagrams demonstrated participants’ individuals interests and perspectives within the community-level drivers of sugar-sweetened beverage consumption. Two examples of the individual diagrams created are presented below in Fig. 1a, b. Following expert review and standardisation of similar variable names, 48 unique variables remained for inclusion in the merged diagram. Individual diagrams are available online (see data availability statement below).

### NA and Decision Making Trial and Evaluation Library (DEMATEL) results

**Fig. 2:**
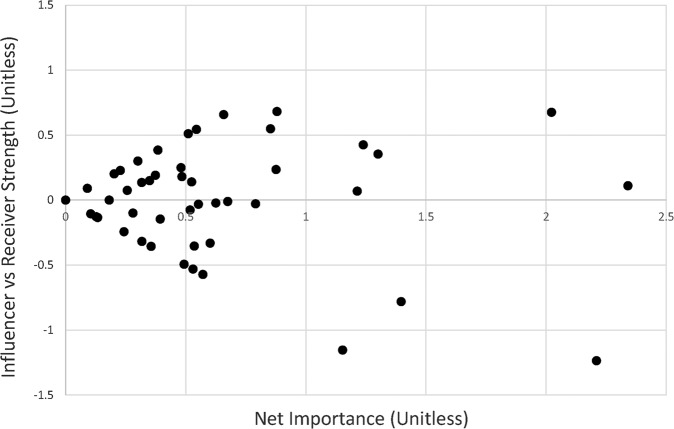
DEMATEL analysis results indicating eight variable cut point. Each variable from the combined diagram is presented on the axes, according to net total importance (*x* axis, unitless) and comparative strength as network influencer vs receiver of influence (*y* axis, unitless). Eight variables are identifiable as a cluster ranging from ~1.15 to ~2.35.

Following application of the NA and DEMATEL analyses, a total of eleven candidates for the converged diagram were generated. Ten candidates were generated using the predetermined 10-variable and 15-variable cut points applied to the four NA rankings, and the DEMATEL ranking. One final candidate was generated based on an 8-variable cut point identified in the clustering within the DEMATEL rankings. The DEMATEL ranking revealed a cluster of strong net influences and receivers of influence within the merged diagram comprising the top eight factors. The DEMATEL results are presented below in Fig. 2, which represents each factor as a point on two axes. The horizontal axis shows the net importance of each variable, equivalent to the sum of its strengths as a network influencer, and receiver of influence within the network. The vertical axis shows the difference between the factors influencer and receiver of influence scores, with a positive score indicating that the variable is an overall influencer, while negative scores indicate an overall receiver. Scoring of the full set of variables from the merged diagram, by analysis technique is available online (see data availability statement).

**Fig. 3:**
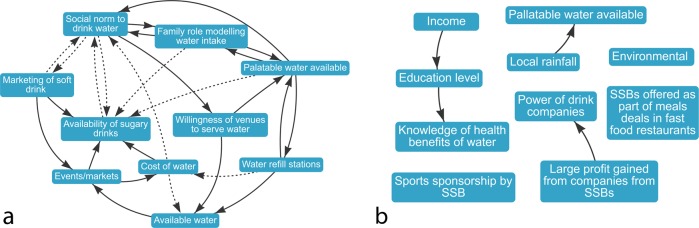
Example of candidate diagrams produced by the pruning analyses. **a** A viable diagram produced by one analysis approach, with factors interconnected in a single unified network of causes and effects, as is typical of a causal diagram. **b** A non-viable diagram, where the analysis selected variables that were not directly connected in the merged diagram. This resulted in a candidate with few causal connections, and little utility in communicating complexity.

The different analytical techniques delivered a wide range of candidate diagrams. Some diagrams were coherently connected as a single unified diagram showing the causal factors as interconnected (Fig. 3a), while others were largely disconnected with little resemblance to a causal diagram (Fig. 3b). Although some of the candidate diagrams were clearly not viable as a meaningful causal diagram, all maps were presented to participants in the validation questionnaire.

Nine participants completed the validation questionnaire, where diagrams were presented in random order for assessment against the validity criteria. Table 1 below shows the mean score for each candidate diagram (listed by analysis technique and cut point) on the four validation items that participants responded to. The far right column gives the summed difference between the actual mean scores for that candidate diagram, compared to the highest mean score in each of the four validation items.

**Table 1:** Analysis techniques ranked by validity items and deviation-from-best-mean score.

Analysis technique (cut point)	Individual mean scores				Diff from sum of best means
	Ease of understanding	Could describe to others	Can see my contributions	Useful for intervention	
DEMATEL (8)	4.1	3.9	2.0	3.7	0.3
Betweenness (10)	4.1	3.8	1.9	3.8	0.4
DEMATEL (15)	4.0	4.0	2.1	3.4	0.5
Degree (10)	4.0	3.9	2.1	3.4	0.6
DEMATEL (10)	3.8	3.9	2.1	3.6	0.6
Eigencentrality (10)	4.0	3.9	1.8	3.7	0.6
Degree (15)	3.6	3.8	1.8	3.6	1.2
Eigencentrality (15)	3.2	3.6	1.6	3.4	2.2
Betweenness (15)	3.3	3.6	1.7	3.1	2.3
Closeness (15)	1.4	2.0	0.7	0.7	9.2
Closeness (10)	−0.1	−0.3	−1.9	−2.2	18.5

**Fig. 4:**
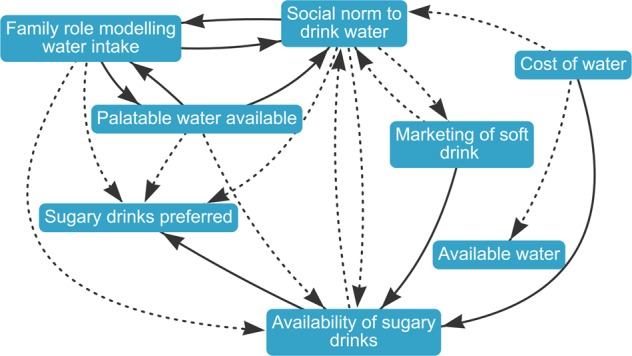
Highest ranked candidate diagram from the validation survey. This diagram shows the set of variables and connections remaining after pruning the merged diagram according to the DEMATEL ranking, with an eight-variable cut point. This map was endorsed most strongly by the users in the validation survey.

The candidate diagram generated using the DEMATEL ranking, with the eight-variable cut point (identified based on the clustering in the analysis results) (Fig. 4) had the lowest total difference (0.3) from the best mean scores across the four indicators, and was therefore considered the preferred candidate among the participants who were surveyed. Other candidates that performed well were the betweenness centrality candidate, with a 10-variable cut point (difference of 0.5), the DEMATEL candidate with a 15-variable cut point 15 (difference of 0.4) and the degree candidate with a 15-variable cut point (difference of 0.4). The full index of candidate diagrams is available online (see data availability statement below).

## Discussion

STICKE provided an accessible, computer-mediated tool for community members to remotely build causal diagrams, using a supported online process. We applied methods to collate, analyse, and synthesise multiple diagrams into a map that summarised multiple participants’ views on the drivers of a complex health problem. Participants identified outputs as easy to interpret and communicate to others, and useful for the purpose of identifying potential interventions.

The online delivery format that STICKE uses is a key strength that enables participatory system mapping activities to reach new participant groups, in support of other community-level systems thinking endeavours. STICKE enables rapid input from community members who may be otherwise difficult to engage due to social isolation, geographical disadvantage, or social or cultural barriers.

This mode of participation also presents potential limitations as other, more formal participatory systems thinking activities such as GMB confer strong benefits attributable to face-to-face group dynamic and engagement, and collaborative discussion.^16,17^ Although STICKE is a very accessible platform with the potential for high reach within communities, participants attending activities such as GMB may see better outcomes in relation to increasing individual understanding of complex problems, and greater ownership and buy-in to community-designed responses to the problem. STICKE therefore should not be taken as a replacement for other rigorous processes such as GMB and other efforts to directly engage and empower marginalised groups. STICKE represents an additional tool that may expand opportunities for community members to reflect on problems from a systems thinking perspective, and benefit from insights about complexity.

The development of STICKE as a tool for broad community engagement in investigating complex problems represents a step beyond current literature in participatory systems mapping methods. Alongside the online computer-mediated system modelling activity supported here, the main activity of STICKE is based on elements of the GMB script literature, meaning it can be used as a facilitation tool within formal GMB workshops, provided the connection circles activity is appropriate for the setting and desired outcomes. In these settings, STICKE could support both formal participatory system modelling efforts in CBPR, and subsequent work to engage broader community with its outcomes via less formal, but still valuable modelling exercises.

This project also demonstrates methods for the collation and synthesis of causal diagrams from multiple community members. Previous published studies synthesising causal diagrams have focussed on the presentation of variable “themes” across multiple diagrams,^18^ or on manual synthesis of models by hand.^19^ The variable theming approach is limited as the analysis removes all consideration of the connections from individual diagrams, simplifying outputs down to lists of similar variables, and removing the critical notion of complexly interconnected determinants that is integral to systems approaches to complex problems. Manual synthesis of individual diagrams overcomes this by producing a converged outcome that retains the form and insights expected from a causal diagram, however in community-based projects the requisite expertise may not be available. This study has demonstrated the utility of the DEMATEL approach in identifying a structural core within a merged set of causal diagrams that users agree is digestable, describable and useful for thinking about intervention, albeit among a smaller initial set of diagrams. DEMATEL is amenable to automation in future iterations of STICKE, which would increase the accessibility of this function.

Alongside the trend toward complexity in population health research,^1,20^ participatory activities that engage complexity and build local engagement are becoming increasingly prominent. Publications have presented case studies of community-led efforts to create systems-informed interventions,^10^ however any community engagement effort will face critical decisions around project reach, and how to balance community engagement against scope and available resources. STICKE provides a community-centric platform that can support systems thinking activities, throughout the community. Supporting communities to tackle complex problems will be critical for sustainable uptake of new approaches to tackling complexity at all scales.

Community-led interventions and CBPR approaches to prevention are among the most promising for impact and sustainability,^21^ and understanding how researchers can best support communities in their intervention efforts will bring more success in population-level prevention going forward.

Although much of the analytic methods described in this study are able to be fully automated, and may be integrated into STICKE in future iterations of the platform, a limiting step in the process was the merging of similar constructs given different names by users. The decision to merge variables often required consideration of the context the variable within its original diagram, and was completed through expert consensus by the research team in this project. The complexity of language and expression means this task remains a challenge, and automating this process was beyond the scope of this project.

The analytic techniques reported here could also be applied to other banks of topic-related diagrams completed by different groups. Application of the DEMATEL analysis to banks of these diagrams could yield interesting insights into common drivers of health and wellbeing across larger aggregated geographical areas, community demographics, or other divisions representing the collective insight of hundreds of individuals across communities.

STICKE is a viable causal diagramming tool that that is easy to use, which may also have the capability to engage broad community groups in insightful systems thinking activities online, and support systems-level change in CBPR.

## Methods

### Study design and setting

Prior to the commencement of the study, sugar-sweetened beverage consumption was selected as the complex issue for consideration among the test users. This issue was chosen as it aligned with existing work considering sugar-sweetened beverage consumption from a systems-thinking perspective among the participant group.

To address the first study aim, STICKE was trialled online to test whether users could successfully complete the computer-mediated activity and produce a causal diagram on the basis of the instructions provided.

To address the second aim, several methods were tested for collating the individual causal diagrams generated by the online participants (described in detail below). To identify which method was most preferred, a survey was designed to seek feedback on the palatability of the various summary diagrams.

### Participants and recruitment

Participants were recruited through health service networks in two regional centres in south-west Victoria, and a university-based mailing list from a university located in a nearby regional centre. The networks in south-west Victoria included health workforce contacts connected to a whole-of-community obesity prevention initiative with CBPR underpinnings. The university-based mailing list included research and teaching staff within a school with a population health focus. Key stakeholders with access to these networks were recruited to act as third-party recruiters, distributing invitations through their networks with support from the research team. Recruiters included health promotion officers in the smaller regional centres, and administrative staff with access to the mailing list within the university setting. These recruitment strategies were intended to engage with participants with diverse professional and academic experience across health-related disciplines.

Participants replied to the researchers in response to the circulated invitation, and received instructions about the study on-line before using STICKE to complete the mapping activity. Participants were contacted again with a follow-up request to complete the validation survey.

### Computer-mediated participatory system mapping activity

The participatory system mapping activity supported by STICKE is based on the connection circles activity, which is documented in the Scriptapedia database (en.wikibooks.org/wiki/Scriptapedia/Connection_Circle), and can be used in concert with other scripts to form part of a formal group model building process.^22^ The connection circles activity is intended as an easy entry point to the creation of a causal diagram via a structured process for recording problem drivers, and connections between those drivers.

**Fig. 5:**
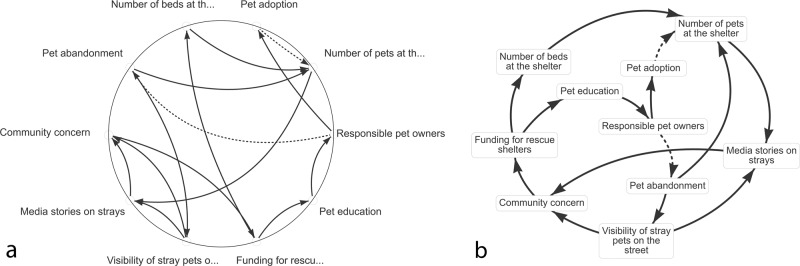
Connection circle and diagram view in the STICKE user interface. **a** The connection circle view as it appears in STICKE following user input of factors deemed important drivers of the problem, and causal relationships between those factors. **b** The diagram view as it appears in STICKE following automated untangling of the connection circle in 5a.

STICKE first presents the user with an empty “connection circle”—which appears to the user as a plain circle on a white background. The user adds variables that they perceive to be drivers of the complex problem under consideration. Following the entry of the problem drivers, the user enters causal relationships between determinants (including the specification of relationship polarity, using system dynamics modelling conventions). The user adds factors and connections in any desired order. STICKE provides a function which automates the process of untangling the connection circle into a more conventional causal diagram. The untangling process is led by a modified force-layout algorithm that analyses model structure and reconfigures the map based on the objectives of minimising crossover of connection arrows, and emphasising feedback loops. The algorithm-driven approach achieves varying levels of “completeness” depending on the number of factors present when switching from connection circle to diagram view, the density of interconnections, and the personal preference of the user. Figure 5 provides examples of the connection circle (5a) and diagram views (5b) in STICKE.

### Causal diagram analysis methods and validation

We trialled multiple analytic methods under the following process: (1) combine all individual causal diagrams into a “merged diagram” that captured every variable and connection proposed by the participants, and (2) identify a theory-based method to prune the merged diagram back to a more concise “converged diagram” that presents useful insights, and allows participants to recognize where their perceived drivers fit against drivers identified by other participants.

#### Merging causal diagrams

Prior to merging individual causal diagrams, variable naming was addressed by standardising the names of variables in different diagrams that were named differently but had similar meaning. For example, one user may name a driver of unhealthy eating as “proximity to fast food”, while another may choose the name “distance to junk food restaurant”. In both cases the intent of the variable is similar and can be assumed to be describing the same basic construct, and can therefore be given a consistent name. The decision to merge and standardise similar variables was completed by expert consensus among the research team, who have research experience facilitating group model building workshops with community groups directly focused on sugar-sweetened beverage consumption, and related problems. Following standardisation of variable names, all individual diagrams were collapsed into one merged diagram that contained the full set of unique variables and connections from each participant.

#### Reducing variables in merged diagram

Multiple analytical techniques were trialled to reduce the large number of variables in the merged causal diagram to a smaller diagram. In the first instance, the reduced causal diagram produced by each analytical technique was referred to as a “candidate diagram”. The goal of the validation survey (explained below) was then to identify the candidate diagram most preferred by the users, which would then be dubbed the “convergent diagram”.

Analytical techniques were taken from the field of NA, which has been previously demonstrated as a tool to investigate important structural elements within causal diagrams,^23,24^ and the DEMATEL, which is an established analytical technique for identifying important components within complex cause-and-effect systems.^25,26^ Negative polarity causal connections were inverted before and after the DEMATEL method. Each analytical technique provided a score for each variable in the merged diagram based on that technique’s analysis of the overall diagram structure. For the NA techniques, variable rankings were used to create reduced candidate diagrams using a 10-variable and 15-variable cut point. In addition to the 10- and 15-variable cutpoints, the DEMATEL approach allows for additional analysis-driven cut points to be identified. DEMATEL provides ranking metrics not only on the overall “importance” of nodes within a network, but also provides metrics on whether they are a net influencer or net receiver of influence within the network. Examination of variable scores using DEMATEL allows for the identification of clusters of strong influencers and receivers of influence within the network, which were used to generate an additional theory-driven cut point.

NA techniques applied to the merged diagrams included: degree (the number of connections to each variable), betweenness centrality (the degree to which a variable act as a pathway between other pairs of variables), eigenvector centrality (connection to other highly connected variables) and closeness (the number of connections required to link from that variable to other variables in the diagrams).

#### User validation of candidate causal diagrams

Following the generation of the candidate causal diagrams, a short validation survey was devised to seek feedback from the users regarding which candidate diagram they preferred. Users were forwarded an email link to complete the survey online. The survey assessed users preference based on ease of understanding, confidence in explaining the diagram to others, ability to identify their own contribution within the diagram, and perceived usefulness in thinking about action.

For each candidate diagram, participants responded to four statements, indicating their agreement or disagreement that they; (1) considered the candidate diagram easy to understand; (2) would be able to describe the content of the candidate diagram to others; (3) could identify their individual contribution within the candidate diagram; and, (4) considered the candidate diagram to be useful for thinking about potential interventions. For each question, participants indicated their agreement or disagreement on an eleven-point Likert scale, from −5 (strongly disagree), through zero (neither agree nor disagree) to +5 (strongly agree).

For each candidate diagram, a mean score for each of the four validation items was calculated. The candidate diagram with the smallest summed difference from the highest mean score on all four validation items was selected as the “user preferred” convergent diagram.

### Ethics

Ethical approval was granted by Deakin University for the completion of this research project. All participants gave written informed consent prior to completing any activities, and organisational consent was secured where third-party recruitment was used. The approval reference for this study is: HEAG-H 113_2015.

### Reporting summary

Further information on research design is available in the Nature Research Reporting Summary linked to this article.

## Supplementary information

Reporting Summary

## Data Availability

The anonymised data that support this study, along with tabulated outputs from all analyses are available publically at: https://deakin365-my.sharepoint.com/:f:/g/personal/josh_hayward_deakin_edu_au/EmRIGg2SURlEjWehDrudQOUBg21n0Q39tIZklapouwDv2Q?e=TahLNR. Further information will be provided upon reasonable request from authors SA and JH.

## Code availability

Standard, publically available coding procedures were used for the Network Analysis and DEMATEL analyses described in this study.
